# Transforming a Pair of Orthogonal tRNA-aminoacyl-tRNA Synthetase from *Archaea* to Function in Mammalian Cells

**DOI:** 10.1371/journal.pone.0011263

**Published:** 2010-06-22

**Authors:** Gabrielle Nina Thibodeaux, Xiang Liang, Kathryn Moncivais, Aiko Umeda, Oded Singer, Lital Alfonta, Zhiwen Jonathan Zhang

**Affiliations:** 1 Division of Medicinal Chemistry, College of Pharmacy, University of Texas at Austin, Austin, Texas, United States of America; 2 Laboratory of Genetics, The Salk Institute for Biological Studies, La Jolla, California, United States of America; 3 Avram and Stella Goldstein-Goren Department of Biotechnology Engineering, Ben-Gurion University of the Negev, Beer-Sheva, Israel; The Scripps Research Institute, United States of America

## Abstract

A previously engineered *Methanocaldococcus jannaschii* 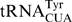
–tyrosyl-tRNA synthetase pair orthogonal to *Escherichia coli* was modified to become orthogonal in mammalian cells. The resulting 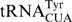-tyrosyl-tRNA synthetase pair was able to suppress an amber codon in the green fluorescent protein, GFP, and in a foldon protein in mammalian cells. The methodology reported here will allow rapid transformation of the much larger collection of existing tyrosyl-tRNA synthetases that were already evolved for the incorporation of an array of over 50 unnatural amino acids into proteins in *Escherichia coli* into proteins in mammalian cells. Thus we will be able to introduce a large array of possibilities for protein modifications in mammalian cells.

## Introduction

There are several different approaches for proteins bearing post-translational modifications. Approaches that make use of expression vectors with classical molecular biology tools for the *in-vivo* expression of proteins are limited to the repertoire of the 20 natural amino acids. Of the 20 natural amino acids, only cysteines (thiol groups) and lysines (amino groups), both of which can be specifically reacted with maleimide or active esters, are used for further protein modifications. However, coupling to natural amino acids occurs at random sites, since multiple target sites usually exist in a protein. In an attempt to overcome this challenge, Yanagawa et al. reported a method for C-teminus specific fluorescence labeling of proteins by using puromycin derivatives [1].

The incorporation of unnatural amino acids (UAAs) affords orthogonal chemistry that will occur only with the UAA. Such incorporation can be achieved via protein chemical synthesis or semi-synthesis, since both approaches allow for the site-specific chemical incorporation of UAAs. Both approaches are, however, limited by the size of the synthesized protein and sometimes even (in the case of semi-synthetic approach) in the location of UAA insertion.

Most proteins undergo post-translational modifications and/or bind a cofactor so as to extend their functional properties. Such considerations render protein study extremely challenging. Thus, site-specific incorporation of unnatural amino acids with desired chemical and physical properties into proteins offers a very useful research tool to not only facilitate the study of proteins but also to generate proteins with novel functions [2]. Modifying proteins in mammalian cells co- and post-translationally is of great importance both for *in vivo* as well as for *in vitro* studies of proteins in cellular mechanisms. Hence, the incorporation of pre-modified unnatural amino acids into mammalian proteins has the potential to greatly assist in such endeavors. Our goal is to create a novel genetic code(s) in mammalian cells that will allow the site-specific incorporation of unnatural amino acids into proteins. Technologies to expand the genetic code have already been developed in *Escherichia coli (E. coli)* [3], yeast [2], [4], [5] and mammalian cells [6]–[10]. However, existing methodologies for the creation of tRNA-aminoacyl-tRNA synthetase pairs for mammalian cells are not efficient, due to the complicated process of evolving tRNA synthetases that recognize unnatural amino acids [6]–[8]. These methods all require for a tRNA synthetase to have evolved to recognize an unnatural amino acid and discriminate against the natural amino acid, which may be very challenging.

In the following report on an approach that enables us to exploit a large (>50) set of already evolved *Methanocaldococcus jannaschii* (*M. jannaschii*) tRNA synthetases in *E. coli* [2], [4] in order to incorporate unnatural amino acids into proteins in mammalian cells, thus alleviating the need for the *in vitro* evolution of the entire set of tRNA synthetases in mammalian cells. For an imported tRNA-synthetase pair to be successfully utilized in a given organism, it must retain its orthogonality, namely, it must be ensured that none of the tRNA synthetases of the host organism can aminoacylate the introduced tRNA and that none of the host organism tRNAs will be amino-acylated by the introduced aminoacyl-tRNA synthetase. We report for the first time on a ‘cut and paste’ approach that allows one to transform both a mutant *M. jannaschii* tyrosyl-tRNA synthetase and a mutant *M. jannaschii* 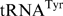 orthogonal to the translational machinery of *E. coli* for use in the site-specific suppression of an amber nonsense codon in genes encoding the green fluorescent protein (GFP) and the foldon protein, both expressed in mammalian cells.

To date and to the best of our knowledge, there are presently four known methodologies to site-specifically incorporate unnatural amino acids into proteins in mammalian cells [6]–[10]. The first takes advantage of a chemically amino-acylated suppressor tRNA, micro-electroporated into mammalian cells, yet is limited by the small amount of protein translated [9]. The second method utilizes an orthogonal tRNA-aminoacyl-tRNA synthetase to site-specifically incorporate unnatural amino acids into a protein. The aminoacyl-tRNA synthetase (RS) is engineered to recognize unnatural amino acids by mutating the active site, based on a known structure [6], [7]. A third approach involves the transplant of a previously evolved tRNA synthetase from *E. coli* and tRNA from *Bacillus stearothermophilus* into mammalian cells to site-specifically incorporate an unnatural amino acid [8]. The final method involves the introduction of a tRNA-aminoacyl-tRNA synthetase pair from *Methanosarcina mazei* in *E. coli* and the shuttling of the pair into mammalian cells, exploiting the unique promiscuity of such pairs and the unique feature of their orthogonality [10].

In the method described here, we utilized an existing *M. jannaschii* synthetase-tRNA pair evolved in *E. coli*, directly in mammalian cells. The advantage of this method is that there is a large pool of evolved *E. coli* synthetase-tRNA pairs for unnatural amino acids already available, as compared to those available in *Saccharomyces cerevisiae*. Our method (detailed below) renders existing methods more accessible by making adaptation quicker and more efficient than in yeast, for example.

## Results and Discussion

To suppress the amber codon inserted into the *gfp* and *foldon* genes introduced into mammalian cells, an orthogonal tRNA synthetase-tRNA pair was first engineered. After the cells were transfected with plasmids encoding this orthogonal tRNA synthetase-tRNA pair, the cellular translation machinery inserted tyrosine into nascent proteins in response to an encountered amber codon within the gene encoding the proteins of interest, namely GFP and foldon. To engineer the orthogonal tRNA synthetase-tRNA pair for subsequent use in mammalian cells, a ‘cut and paste’ approach was utilized. Archaeal and eukaryotic tRNA synthetase-tRNA pairs recognize each other by identity determinants that distinguish them from bacterial tRNA synthetase-tRNA pairs. The key determinant in the archaeal and eukaryal complexes is a single base pair in the tRNA moiety which is recognized by the connective polypeptide region (CP1) in the corresponding tRNA synthetase. Specifically, archaeal/eukaryotic tRNA synthetases recognize the C1:G72 base pair, while bacterial tRNA synthetases recognize the G1:C72 base pair [11]–[13]. The ‘cut and paste’ approach exploits these identity determinants in order to generate an orthogonal tRNA synthetase-tRNA pair in mammalian cells [11], [13], [14]. This novel ‘cut and paste’ method thus allows for rapid engineering of existing evolved *M. jannaschii* tRNA synthetase-tRNA pairs. *M. jannaschii* tyrosyl tRNA synthetase (TyrRS) is an ideal synthetase for such manipulation as it is distinct from the TyrRS's of other organisms since it only uses information found in the 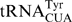 acceptor stem to identify and bind its cognate tRNA [13]. TyrRS from most other organisms also rely on sequence information found in the anticodon loop and D stems [13].

**Figure 1:**
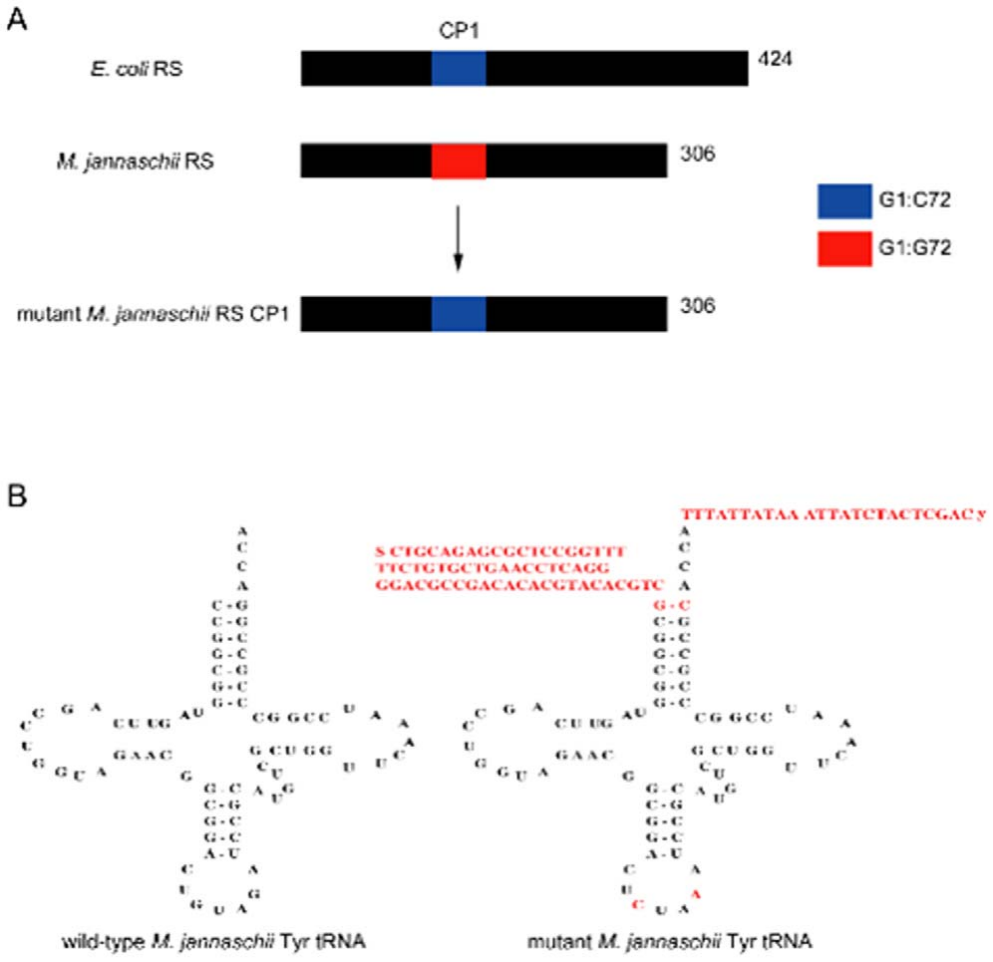
‘Cut and Paste’ approach used to construct an orthogonal *M. jannaschii* tRNA synthetase-tRNA pair for use in mammalian cells. (A) The *M. jannaschii* tRNA synthetase (RS) CP1 region (red) was replaced with the *E. coli* RS CP1 region (blue) to create mutant *M. jannaschii* RS CP1. (B) The mutations in the *M. jannaschii* tyrosyl tRNA mutant are shown in red.

Two critical elements are exploited in pairing *M. jannaschii* TyrRS with its cognate 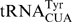, namely a thirty-nine amino acid long connective polypeptide region (CP1) in the TyrRS and a single base pair, C1:G72, found in the acceptor stem of the 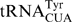. These elements are, however, also found in mammalian synthetases and tRNAs, rendering the *M. jannaschii* TyrRS-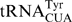 pair non-orthogonal in mammalian cells [11], [13]. Therefore, in order to use *M. jannaschii* TyrRS and 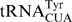 orthogonally in a mammalian system, these sequence elements were altered to their bacterial versions, which are not recognized by eukaryotes (Figure 1). These minimal manipulations had little or no effect on the amino acid selectivity of TyrRS, since the CP1 region of TyrRS is remote from the amino acid-binding site [12]. Next, the base pair, C1:G72 of *M. jannaschii* 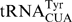 was mutated to G1:C72 to allow the *E. coli* CP1 region in *M. jannaschii* TyrRS to recognize only this specific tRNA. In order for the modified *M. jannaschii* 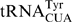 to insert tyrosines at specific sites in GFP, the corresponding anticodon was mutated to CUA, which recognizes its cognate codon (TAG) in mRNA encoding for the GFP protein. Vectors with six tandemly arranged tRNA genes, as well as with a single tRNA gene and the corresponding 5′ and 3′ flanking sequences, were generated, based on previous findings [6].

**Figure 2:**
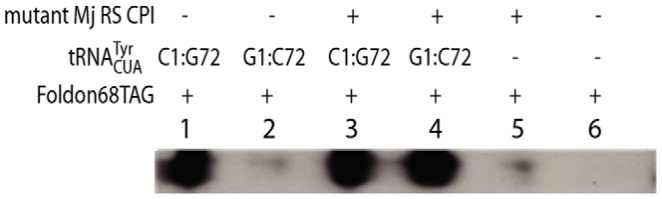
The orthogonality of the *M. jannaschii* TyrRS-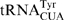 pair was verified on western blots probed with anti-V5 antibodies. Expression of full-length foldon was monitored when various tRNAs were introduced into the HEK 293T cells. Note that the tRNA mutants used in these experiments were slightly different from those depicted in figure 1.

To examine whether our hypothesis regarding the CP1 region, namely that the ‘cut and paste’ approach is significant enough to render orthogonality between *M. janaschii* and mammalian cells, an amber codon was introduced within the *foldon* gene at position 68. The orthogonality of the *M. jannaschii* tRNA synthetase-tRNA pair was observed via western blot. Cells from the HEK 293T cell line were transfected with different combinations of plasmids. All of the cells were also transfected with a plasmid harboring the 68TAG *foldon* mutant. Forty eight hours following transfection, the cells were harvested and foldon was purified, if fully expressed, via affinity purification resin with anti-V5 antibodies. (V5 is a short peptide that was fused to the C-terminal of foldon as an epitope tag). Purified foldon mutants were also subjected to western blot using anti-V5 antibodies. Lanes 1 and 3 of figure 2 it respectively show full length foldon expressed both in the presence or the absence of the mutant *M. janaschii* RS where the CP1 region was switched to recognize the G1:C72 tRNA base pair. These results indicate that the *M. janaschii* C1:G72 tRNA is aminoacylated by endogenous RSs in the mammalian cells. However, there is hardly any detectible mutant foldon when the mutant *M. janaschii* RS is absent (lane 2), yet full length expression when it is present (lane 4). These results are indicative of the fact that the G1:C72 tRNA was not aminoacylated by endogenous RSs, whereas when the mutant RS is present, the tRNA is charged with tyrosine. Further controls demonstrated that in the absence of the mutant suppressor tRNA, no full length foldon was observed, implying that there are no endogenous tRNAs that are charged by the mutant *M. janaschii* RS (lane5) nor that suppression spontaneously occurred within these cells (lane 6). Hence, figure 2 confirms that the single base pair switch in the mutant tRNA together with the CP1 switch in the RS, are sufficient to switch the archaeal pair from being orthogonal in bacteria to being orthogonal in eukaryotes.

**Figure 3:**
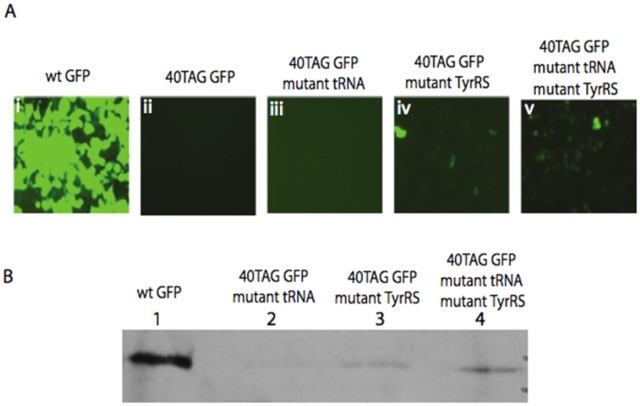
Suppression of an amber codon inserted into the GFP-encoding gene. (A) Full-length GFP was expressed in HEK 293T cells only in the presence of a *M. jannaschii* TyrRS-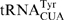 pair designed to be orthogonal to mammalian cells. (B) Western blot analysis of full-length GFP probed with anti-His antibodies.

Next, we wanted to confirm that the observed orthogonality was not protein-dependent or signal-dependent. Accordingly, we assessed the expression of a different protein. Again, HEK 293T cells were transfected to express different combinations of plasmids harboring the *gfp* gene, mutant suppressor 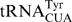 and mutant TyrRS. Mutant GFP protein was encoded by a gene in which the TAG stop codon was inserted at position 40, yielding mutant 40TAGgfp. A construct including wild type gfp (wt gfp) served as a control. The GFP protein, either mutant or wt, was fused to a 6xHis tag at its C-terminal. After transfection, cells were monitored at 24, 48, and 72 hours by fluorescence microscopy for the detection of GFP fluorescence. After 72 hours, pictures of the cells were taken and are shown in the different panels of figure 3A (panels i–v). HEK 293T cells expressed the full-length wt GFP with an efficiency of 95% (figure 3A, panel i). However, we did not observe fluorescence in the absence of the introduced orthogonal pair (figure 3A, ii). When mutant tRNA and mutant GFP were present in the HEK 293T cells, marginal (<1%) fluorescence was detected (figure 3A, iii). The results thus suggest that 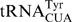 6 (which contains 6 consecutive copies of the mutant tRNAs, shown in figure 1) interacts with endogenous tRNA synthetases only to a small extent. To investigate the orthogonality of endogenous mammalian tRNAs towards the mutant tRNA synthetases, the mutant tRNA synthetase was expressed together with 40TAGGFP. When mutant *gfp* and mutant TyrRS were present in the HEK 293T cells (figure 3A, panel iv), the extent of GFP fluorescence was roughly 5%, as compared to the extent of wt GFP fluorescence. The background signal observed in panel iv (figure 3A) is likely due to the mutant Tyr tRNA synthetase interacting with endogenous tRNAs. Finally, panel v displays fluorescence originating from the suppression assay where plasmids encoding mutant GFP, mutant TyrRS, and mutant 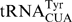 6 were all introduced into the HEK 293T cells. Since plasmid encoding for the mutant GFP was co-transfected with plasmid encoding the newly engineered *M. jannaschii* TyrRS-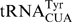 pair, GFP fluorescence (panel v) was roughly 20% of that obtained with wt GFP (panel i). While some stop codon suppression was observed in the negative controls, our results indicate that the ‘cut and paste’ method employed is a valid method for suppressing amber codons in *gfp*. To verify full length GFP expression, all plates were harvested at 72 hours, after which time the cells were lysed, GFP protein was purified and examined by western blot analysis using anti-His antibodies (figure 3B). A strong band of 28 kDa was detected, corresponding to the expression of wt GFP (lane 1). Negative controls using either mutant 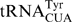 6 or mutant TyrRS designed to assay orthogonality either yielded no antibody-stained band (lane 2) or only a faint band (lane 3), since no more than 5% of full-length GFP was detected in the corresponding fluorescence image (figure 3A, iii and iv respectively) under these conditions. Although a peptide from the N-terminus of GFP (4.4 kDa) is likely to be expressed in the negative control (lane 2), the truncated GFP products are not detected by the western blot because the 6xHis epitope is located at the C-terminal of the protein. When one considers band observed in lane 4 it is clear that suppression of the amber codon in GFP is clearly stimulated (∼20% efficiency), relative to the negative controls containing either mutant 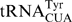 6 or mutant TyrRS. The 20% tyrosine suppression efficiency observed is similar to what has been reported elsewhere, where 20–40% suppression efficiency were typically achieved [15]–[17]. The data shown in figure 3 confirm our hypothesis that ‘cut and paste’ of the CP1 region within the tRNA synthetase is a valid method to rapidly engineer *M. jannaschii* TyrRS-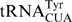 pairs designed to fit into mammalian systems. Our study also provides the first *in vivo* evidence of discrimination in the CP1 region and the G1:C72 or C1:G72 recognition sequence in cognate tRNAs along kingdom lines.

### Conclusions

In conclusion, we have demonstrated that the novel methodology introduced here can be readily applied to the plethora of extant evolved *E. coli* synthetase-tRNA pairs by introducing a few minor changes into the tRNA and synthetase gene sequences. This methodology can thus be used for the incorporation of unnatural amino acids into mammalian proteins, thereby aiding in the study of protein structure and function.

## Materials and Methods

### Strains and Enzymes

Chemically competent *E. coli* TOP10 cells were used for propagation and isolation of all plasmids. Human embryonic kidney 293T (HEK 293T) cells were used for genetic assays. All enzymes were obtained from New England Biolabs (Ipswich, MA), unless otherwise stated.

### Plasmid Construction

DNA encoding wild type (wt) *M. jannaschii* tyrosine-tRNA synthetase (MjTyrRS) was amplified by polymerase chain reaction (PCR) and cloned into plasmid pEF6-V5-His6-TOPO (Invitogen, Carlsbad, CA) according to the supplier's instructions. The connective polypeptide 1 (CP1) region (amino acids 110–148) of MjTyrRS was replaced with the CP1 region of *E. coli* TyrRS (amino acids 110–172). Briefly, the 5′ region of MjTyrRS was amplified using PCR with the primers, EYRS1-topBamH1, (bold letters represent restriction sites) (5′-AA**GGATCC**ACCATGGACGAATTTGAAATGAT-3′) and EYRS1-bot (5′-ACATTCATATTGCCGAACCACTGGAATTCACTTCCAT-3′). The 3′ region of MjTyrRS was PCR amplified using the primers, EYRS3-top (5′-AGGGGATTTCGTTCACTGAGGTTATCTATCCAATAATGCA-3′) and EYRS3-botEcoR1 (5′-CCC**GAATTC**TAATCTCTTTCTAATTGGCT-3′). The *E. coli* CP1 region was PCR amplified from the *E. coli* TyrRS using primers EYRS2-top (5′-ATGGAAGTGAATTCCAGTG GTTC GGCAATATGAATGT-3′) and EYRS2-bot (5′-TGCATTATTGGATAGATAACTTCAGTGAACGAAATCCCCT-3′). The resulting three PCR fragments (i.e. *M. jannaschii* 5′ and 3′ regions, and *E. coli* CP1 region, 1 µg each) were combined, denatured for 15 minutes at 85°C, and elongated at room temperature with Klenow enzyme for 30 minutes, according to the product manual. The Klenow product was PCR amplified using EYRS1-topBAMH1 and EYRS3-botEcoR1 primers to create the full-length *M. jannaschii* TyrRS CP1 mutant. The mutant TyrRS gene and the pEF6/V5 vector (Invitrogen, Carlsbad, CA) were digested with HindIII and EcoRI and the digested products were ligated to yield plasmid pMUTyrRS.

To generate the desired amber suppressor, 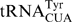, in mammalian cells, overlapping oligonucleotides (restriction sites are in bold) (AA**CTGCAG**AGCGCTCCGGTTTTTCTGTGCTGAACCTCAGGGGACGCCGACACACGTACACGTCGCGGCGGTAGTTCAGCCTGGTAGAACGGCGGACTCTAAATCCGCATG) and (CCG**CTCGAG**TAGATAATTTATAATAAATGGTGCGGCGGGCCGGATTTGAACCAGCGACATGCGGATTTAGAGTCCGCCGTTCTACCA) were designed for the *M. jannaschii* tRNA^Tyr^ sequence with a few modifications. These modifications included altering C1:G72 in the acceptor stem to G1:C72, altering the Tyr anticodon (GUA) to recognize the amber stop codon (CUA), and mutation G38 to A38. In addition, the human tRNA^Tyr^ 5′-flanking sequence (AGCGCTCCGGTTTTTCTGTGCTGAACCTCAGGGGACGCCGACACACGTACACGTC) and a 3′-thymine (T)-rich flanking sequence (TTTATTATAAATTATCTA) were added to the *M. jannaschii* tRNA^Tyr^. The oligonucleotides were annealed and elongated with Klenow polymerase, as described above. The mutant sequence was amplified by PCR, digested overnight with PstI and XhoI, and ligated with T4 DNA ligase into the PstI and XhoI pre-digested plasmid, pZeoSV2(+) (Invitrogen, Carlsbad, CA), to yield pMU 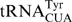. This plasmid was then used as a template for the six unidirectional copy gene cluster. To generate the cluster, mutant *M. jannaschii* tRNA was first amplified by PCR with primers KpnI 1-1 Fwd (GG**GGTACC**AGCGCTCCGGTTTTTCTGTG) and KpnI 1-1 Rev (GG**GGTACC**TAGATAATTTATAATAAATGGTGCGGCGGG). The PCR product, i.e. mutant *M. jannaschii* 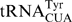, was digested with KpnI overnight, purified with a QIAprep Miniprep Kit according to the manufacturer's instructions (Qiagen, Valencia, CA), and ligated with T4 DNA ligase into the KpnI pre-digested pMU 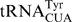 plasmid to yield plasmid pMU 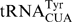 2. The *M. jannaschii* tRNA single copy mutant was PCR amplified with primers EcoRI 1-1 Fwd (G**GAATTC**AGCGCTCCGGTTTTTCTGTG) and EcoRI 1-1 Rev (G**GAATTC**TAGATAAATTTATAATAAATGGTGCGGCGGG). The resulting PCR product was digested with EcoRI overnight and purified with the QIAprep Miniprep Kit. The purified and digested PCR product was ligated in the EcoRI site of plasmid pMU 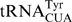 2 with T4 DNA ligase to yield plasmid pMU 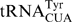 3. Finally, the three copy mutant *M. jannaschii* 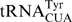 was PCR amplified from plasmid pMU 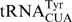 3 using primers NheI 1-1-3 Fwd (GGG**GCTAGC**TTAAGCTTGGTACCAGCGC) and HindIII 1-1-3 Rev (GGG**AAGCTT**GGGCCCTCTAGACTCGAG). The tRNA was digested with NheI and HindIII overnight, purified with the QIAprep Miniprep, and ligated with T4 DNA ligase into pre-digested plasmid pMU 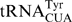 3 to generate plasmid pMU 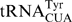 6. The correct construction of plasmid pMU 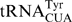 6 was confirmed by DNA sequencing (University of Texas Core Facility).

The codon for Tyr40 of the green fluorescent protein (GFP) gene under control of a CMV promoter in a mammalian expression vector (pLit) was mutated to an amber codon (TAG) using the Quikchange II Site-Directed Mutagenesis Kit (Stratagene, La Jolla, CA), as directed. The identity of the resulting p40TAGGFP plasmid was confirmed by DNA sequencing (University of Texas Core Facility).

The pFoldon plasmid, which was used to express the bacteriophage T4 fibritin (foldon) domain in HEK 293T cells, was constructed by inserting the PCR-amplified gene fragment into the pCDA3.1-V5-His-TOPO vector (Invitrogen, Carlsbad, CA). Plasmid pFoldonTAG, which encodes the Trp68TAGfoldon mutant, was constructed by site directed mutagenesis by using the QuikChangeXL (Staratagene, La Jolla, CA) method and the corresponding HPLC-purified primers.

### Cell Culture and Transfections

HEK 293T cells ATCC® CRL-11268™ were cultured in Dulbecco's Modified Eagle Medium (D-MEM) high glucose (Invitrogen, Carlsbad, CA) supplemented with 10% (v/v) heat-inactivated Fetal Bovine Serum (FBS) Premium Select, Lot # C0136 (Atlanta Biologicals, Lawrenceville, GA) and 1% penicillin-streptomycin (HyClone, Logan, UT) at 37°C in a humidified incubator containing 5% CO_2_. Transfections were performed at 70–80% confluency in a 100-mm plate (Corning, Corning, NY) using FuGENE 6 (Lot # 93576920; Roche, Indianapolis, IN). The pMUTyrRS, pMU 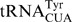 6, and p40TAGGFP or pFoldonTAG constructs were transiently transfected into HEK 293T cells, as described in the FuGENE 6 manual. Full-length GFP was excited at 460–500 nm and detected in the cells by monitoring emission wavelength (510–560 nm) using a Nikon Eclipse TE2000-S microscope equipped with a FITC HyQ filter (Chroma, Rockingham, VT). Negative controls lacking each of the aforementioned constructs, and positive controls transfected to express wild type GFP were also simultaneously generated. All genetic experiments were performed in triplicate.

### Harvest and Lysis of HEK 293T cells

The mammalian plasmids p40TAGGFP and pLit harboring the gene that encodes GFP containing a 6x-histidine tag to facilitate protein purification. Plasmid pFoldonTAG harbors the *foldon* gene and encodes for a V5 peptide tag to facilitate its affinity purification using an anti-V5 antibody-bearing agarose affinity gel (Sigma, Saint Louis, MO). The HEK 293T cells were harvested in 1× PBS, after being physically scraped from growth plates and centrifuged at 1000×g. The collected cells were re-suspended in 2.5× passive lysis buffer (Promega, Madison, WI) supplemented with a Complete Mini-Protease inhibitor tablet (Roche), and incubated on ice for 30 minutes. The lysed cells were centrifuged to collect the soluble protein fraction, which was subsequently visualized by western blot.

### Western Blots

Full-length mammalian-derived GFP or foldon found in the supernatant collected above were separated by 12% sodium dodecylsulphate-polyacrylamide gel electrophoresis (SDS-PAGE) and transferred onto an Immobilon PVDF membrane (Millipore, Billerica, MA) in transfer buffer (48 mM Tris, 39 mM glycine, 20% methanol (v/v), pH 9.2) using a BioRad Semi-Dry Blotter. The membrane was then probed with anti-V5 C-terminal primary antibodies (Invitrogen, Carlsbad, CA) and a goat anti-mouse alkaline phosphatase conjugated secondary antibodies (BioRad, Hercules, CA) or with anti 6xHis C-terminal antibodies (Invitrogen, Carlsbad, CA). The bands were visualized with a chemiluminscent reagent, PhosphaGlo AP (KPL, Gaithersburg, MD), on Kodak BioMax Light Film. The western blots experiments were repeated no less than three times.
